# Echinoderm radial glia in adult cell renewal, indeterminate growth, and regeneration

**DOI:** 10.3389/fncir.2023.1258370

**Published:** 2023-09-29

**Authors:** Vladimir Mashanov, Soji Ademiluyi, Denis Jacob Machado, Robert Reid, Daniel Janies

**Affiliations:** ^1^Wake Forest Institute for Regenerative Medicine, Winston-Salem, NC, United States; ^2^Department of Bioinformatics and Genomics, College of Computing and Informatics, University of North Carolina at Charlotte, Charlotte, NC, United States

**Keywords:** echinoderm, radial glia, neurogenesis, regeneration, indeterminate growth, Myc

## Abstract

Echinoderms are a phylum of marine deterostomes with a range of interesting biological features. One remarkable ability is their impressive capacity to regenerate most of their adult tissues, including the central nervous system (CNS). The research community has accumulated data that demonstrates that, in spite of the pentaradial adult body plan, echinoderms share deep similarities with their bilateral sister taxa such as hemichordates and chordates. Some of the new data reveal the complexity of the nervous system in echinoderms. In terms of the cellular architecture, one of the traits that is shared between the CNS of echinoderms and chordates is the presence of radial glia. In chordates, these cells act as the main progenitor population in CNS development. In mammals, radial glia are spent in embryogenesis and are no longer present in adults, being replaced with other neural cell types. In non-mammalian chordates, they are still detected in the mature CNS along with other types of glia. In echinoderms, radial glia also persist into the adulthood, but unlike in chordates, it is the only known glial cell type that is present in the fully developed CNS. The echinoderm radial glia is a multifunctional cell type. Radial glia forms the supporting scaffold of the neuroepithelium, exhibits secretory activity, clears up dying or damaged cells by phagocytosis, and, most importantly, acts as a major progenitor cell population. The latter function is critical for the outstanding developmental plasticity of the adult echinoderm CNS, including physiological cell turnover, indeterminate growth, and a remarkable capacity to regenerate major parts following autotomy or traumatic injury. In this review we summarize the current knowledge on the organization and function of the echinoderm radial glia, with a focus on the role of this cell type in adult neurogenesis.

## 1. Introduction

**Figure 1:**
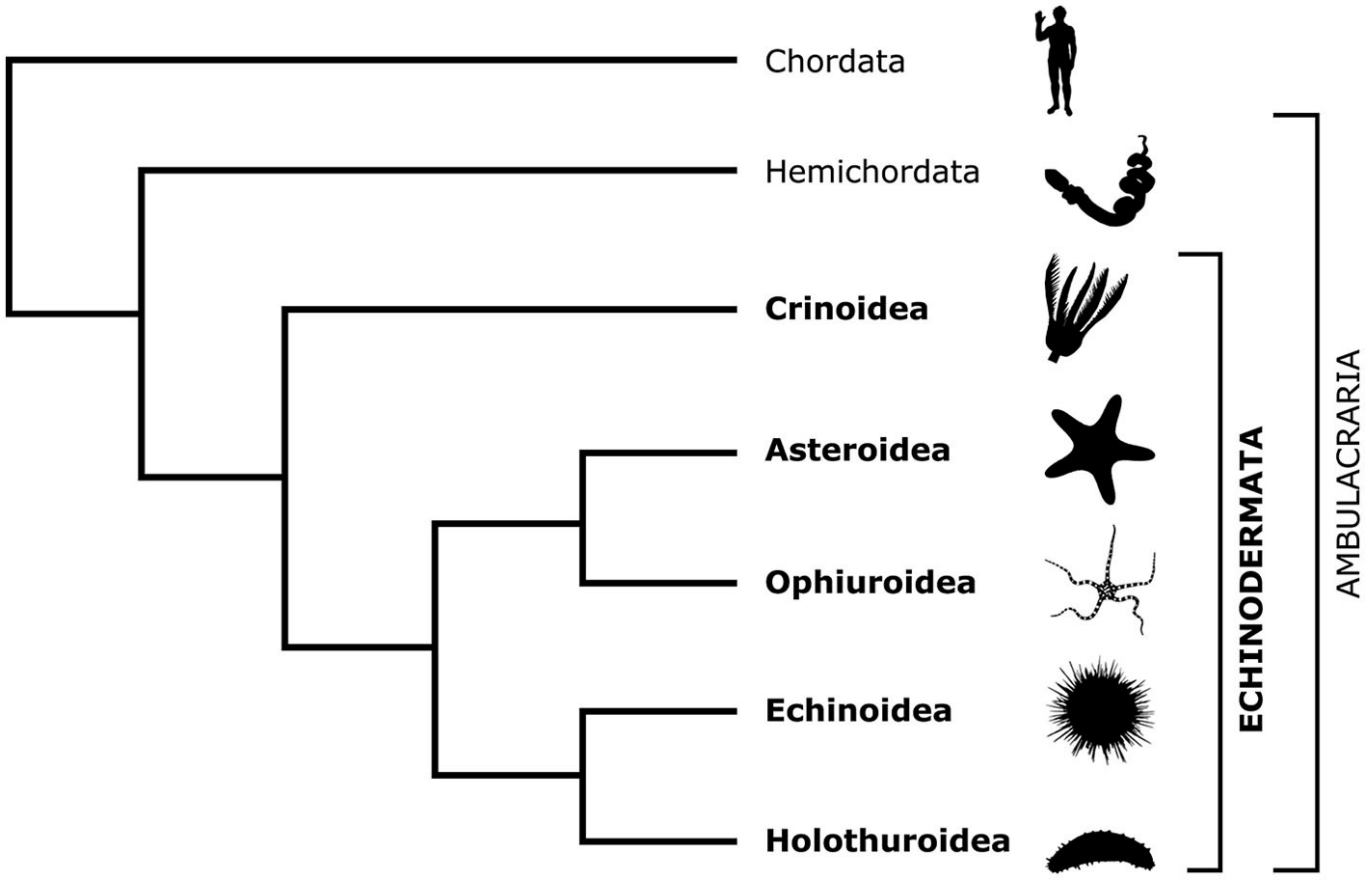
Cladogram showing the phylogenetic position of the phylum Echinodermata in relation to Chordata and Hemichordata and the relationships between the five extant echinoderm classes. The silhouette images are from PhyloPic (https://www.phylopic.org/).

Echinoderms are a phylum of exclusively marine invertebrates, whose biology is fascinating at many levels. Phylogenetically, echinoderms are placed, together with hemichordates, as a sister taxon (Ambulacraria) to chordates within the monophyletic group Deuterostomia (Figure 1). This deep common ancestry with our own phylum makes echinoderms particularly interesting, informative, and relevant to fundamental science and translational medicine. The importance of echinoderm research for fundamental science is due to the insights it provides into the evolution of organ systems, body plans, and developmental trajectories (Heinzeller and Welsch, 2001; Swalla, 2006; Lowe et al., 2015; Adameyko, 2023; Nanglu et al., 2023). The value of echinoderm studies in translational medicine is due to the unusually high regenerative capacities of adult echinoderm tissues, including the central nervous system (CNS).

**Figure 2:**
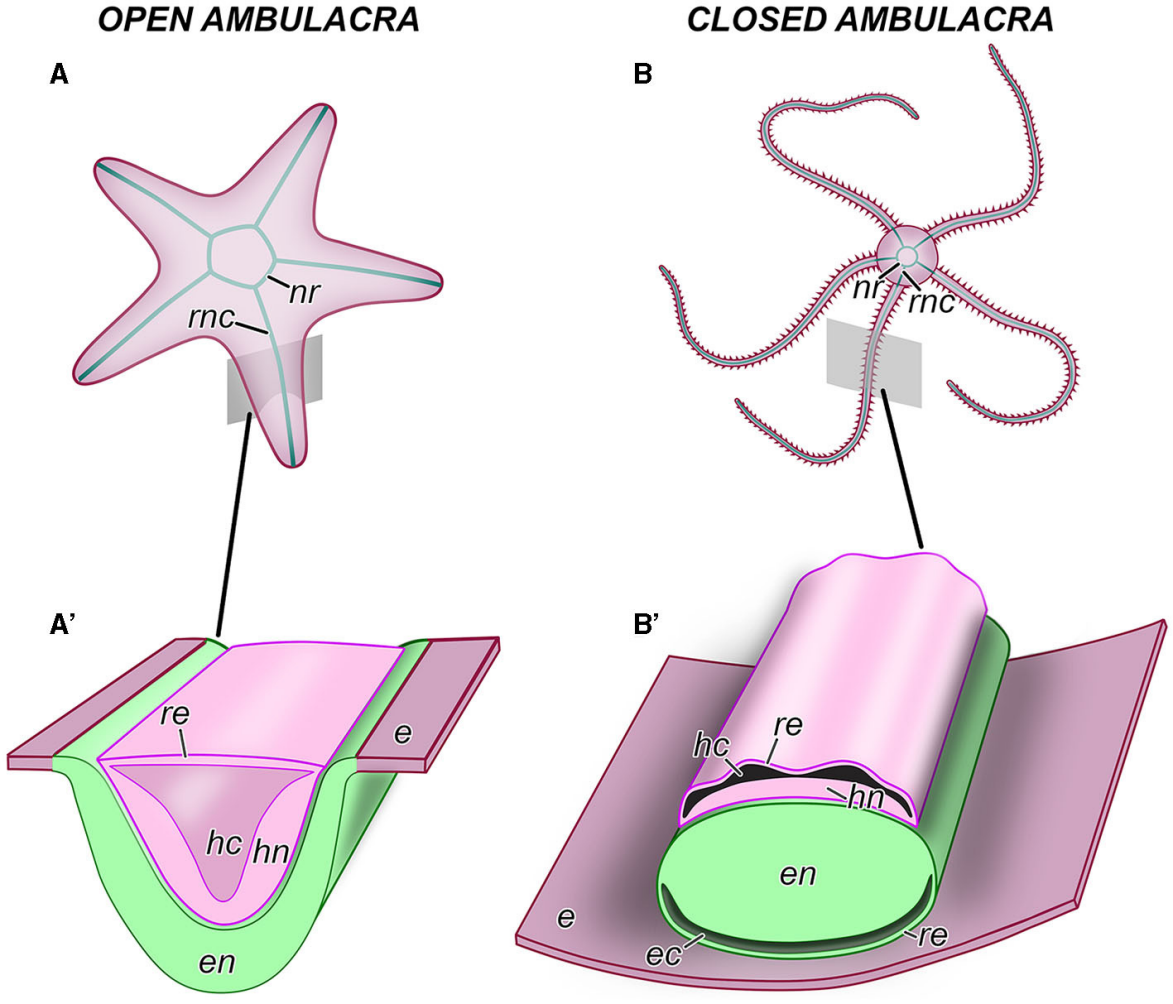
Anatomical organization of the central nervous system in echinoderms. **(A, A')** An example of an echinoderm with the open ambulacra (an asteroid). **(B, B')** An example of an echinoderm with closed ambulacra (an ophiuroid). **(A, B)** General anatomy of the CNS, aboral view. **(A', B')** Diagram of the cross section through the oral region of an arm showing the organization of the radial nerve cord. *e*, oral epidermis; *ec*, epineural canal; *en*, ectoneural neuroepithelium; *hc*, hyponeural canal; *hn*, hyponeural neuroepithelium; *nr*, nerve ring; *re*, roof epithelium; *rnc*, radial nerve cord.

There are about 7,000 extant echinoderm species (Satoh, 2016) that are classified into five classes: Crinoidea (sea lilies and feather stars), Asteroidea (starfish), Ophiuroidea (brittle stars), Echinoidea (sea urchins), and Holothuroidea (sea cucumbers; Figure 1). Unlike other deuterostomes, modern echinoderms have secondary pentaradial symmetry, with five body axes converging at the mouth (Figure 2). In stellate forms, which include crinoids, asteroids, and ophiuroids, these five axes form body extensions called arms. In globose forms—echinoids and holothuroids—these axes run between the oral and aboral poles of the body and subdivide it into five sectors.

**Figure 3:**
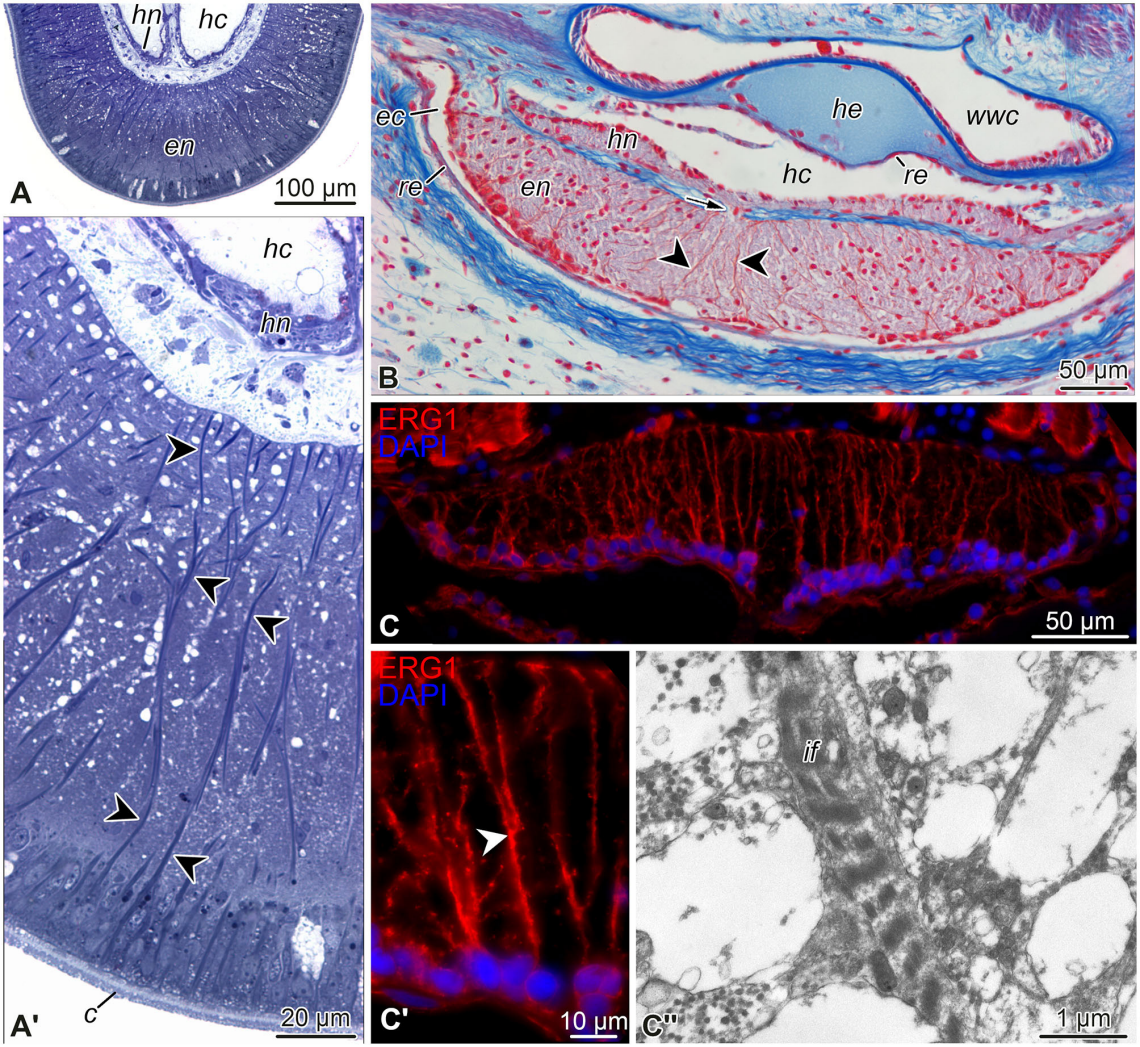
Representative micrographs showing radial glial cells in the echinoderm nervous system. **(A, A')** Semi-thin (0.8 μm) transverse plastic sections of the radial nerve cord in the starfish *Aphelasterias japonica*. Methylene blue. **(A)** General view of the radial nerve cord. **(A')** Higher magnification view of the ectoneural neuroepithelium. **(B)** Paraffin cross-section through the radial nerve cord and adjacent anatomical structures of the sea cucumber *Eupentacta fraudatrix*. Heindenhein's azan. **(C–C”)** Radial glia in the radial nerve cord of the brittle star *Ophioderma brevispinum*. **(C, C')** General view **(C)** and higher magnification **(C')** of the radial nerve cord immunostained with the radial glia-specific antibody ERG1 (Mashanov et al., 2010). Nuclei were stained with DAPI. **(C”)** Transmission electron micrograph of intermediate filaments in a radial glia basal process in the ectoneural neuropil. *Arrow* (in **B**) shows a short neural bridge connecting the ectoneural and hyponeural parts of the radial nerve cord. *Arrowheads* in **(A', B, C')** indicate basal processes of radial glial cells. *c*, cuticle; *ec*, epineural canal; *en*, ectoneural neuroepithelium; *hc*, hyponeural canal; *he*, radial hemal lacuna; *hn*, hyponeural neuroepithelium; *if* , intermediate filaments; *re*, roof epithelium; *wwc*, radial canal of the water-vascular system.

The gross anatomical organization of the echinoderm CNS follows the pentaradial design of the body plan. Each axis is supplied with a *radial nerve cord (RNC)*, which is accompanied by other radial organs, including the hemal canal, the water-vascular canal, and muscle(s) (Figures 2, 3A–C). In the vicinity of the mouth, the five RNCs are joined together by a *circumoral nerve ring* (Figure 2). Because of its pentaradial design, the echinoderm CNS had been considered for decades to be too “derived” and “enigmatic” (Cobb, 1989), with unclear homology relationships to its counterparts in other deuterostome animals and bilaterians in general. As a result, research into the neurobiology of echinoderm has made much slower progress compared to that in other phyla. Fortunately, the situation has changed in the last two decades due to a serious effort to resolve at least some of the homology issues. The problem of homologizing the anatomical components of the echinoderm CNS to those of other deuterostomes is contingent on resolving the homology of the main body axes in the adult pentaradial echinoderm body. Axis comparison between echinoderms and other deuterostomes is one of the most problematic outstanding issues in the modern evolutionary developmental biology (Heinzeller and Welsch, 2001; Swalla, 2006; Lowe et al., 2015; Adameyko, 2023; Nanglu et al., 2023). What makes this conundrum particularly difficult to resolve is the challenge of synthesizing and interpreting the growing body of paleontological, embryological, and molecular data.

Although not yet universally accepted (Adachi et al., 2018; Formery et al., 2023), one school of thought, to which we adhere, postulates that the ambulacra (or arms in stellate forms) of echinoderms can be at least partially homologous to the chordate trunks (Heinzeller and Welsch, 2001; Byrne et al., 2016; Nanglu et al., 2023). It then logically follows that the pentamery that is the hallmark of the modern echinoderm body plan has arisen via multiplication of the single ancestral anterior-posterior axis with the five radii arranged around the shared mouth. Further, within the framework of this hypothesis, each of the echinoderm radial nerve cords would be homologous to the chordate neural tube. This hypothesis is well-supported by the paleontological data, which shows gradual acquisition of the pentaradial body plan from the bilateral design through a series of intermediate stages. The most basal echinoderms (e.g., *Ctenoimbricata* and ctenocystoids) were bilateral, with a clearly defined anterior-posterior body axis (Zamora et al., 2012; Zamora and Rahman, 2014). Later pre-radial groups, such as stylophorans and solutes, had a well-developed bilaterally symmetrical single segmented arm thought to be supplied with a canal of the water-vascular system and a single radial nerve (Lefebvre et al., 2019; Clark et al., 2020). The multiplication of the original single body axis occurred in several Cambrian echinoderms: cinctans had two ambulacra (Zamora and Smith, 2008), whereas helicoplacoids had three radii incorporated into their body wall (Zamora and Rahman, 2014). The pentaradial symmetry first appeared in helicocystoids and then became a fixed feature in the crown-group echinoderms (Zamora and Rahman, 2014). The step-wise acquisition of pentamery seen in early echinoderm evolution is “recapitulated” in the CNS development of some echinoderms. For example, in the sea cucumber *Eupentacta fraudatrix* (Mashanov et al., 2007), the earliest CNS rudiment has a single (mid-ventral) radial nerve cord and thus displays clear bilateral symmetry. In older individuals, two more cords (dorsal) appear simultaneously, thus marking a transition to the tri-radial symmetry. Finally, the pentaradial symmetry is established when a pair of ventral lateral cords grow from the nerve ring.

Another impetus for the recent progress in echinoderm neurobiology is due to the renewed interest in post-traumatic neurogenesis. One of the most interesting developments in the recent years has been the realization that echinoderms possess radial glial cells in the CNS (Mashanov et al., 2006, 2009, 2010; Zueva et al., 2018). Furthermore, there are similarities in functions of radial glia in echinoderms and chordates, including their role as a major progenitor population in neurogenesis (Mashanov et al., 2008, 2010, 2013, 2015b,c, 2022).

In this review, we summarize the current knowledge on echinoderm radial glia, highlight the gaps that still exist in our understanding, and outline the avenues for future research.

## 2. Anatomical organization of the echinoderm CNS

As the organization of the echinoderm CNS is not part of the common knowledge among non-specialists, we provide a brief anatomical overview and introduce the necessary terminology here. For more detailed accounts, the reader is referred to the classical and more recently published reviews (Hyman, 1955; Mashanov et al., 2016). As in other bilaterians, the echinoderm nervous system is composed of the CNS and peripheral nerves, with the latter connecting the former to effectors and sensory organs.

As introduced above, the CNS includes five radial nerve cords that are joined together by a circumoral nerve ring (Figures 2A, B). At the microanatomical level, the CNS is composed of two closely apposed, but distinct layers of nervous tissue called the *ectoneural* and *hyponeural systems* (Figures 2A', B', 3A, A', B). The anatomical location of the ectoneural layer varies among the five echinoderm classes. In crinoids and asteroids, it is located on the oral surface of the body (Figures 2A', 3A, A'). These are the animals with open ambulacra. In contrast, the three remaining classes (i.e., ophiuroids, echinoids, and holothuroids) have closed ambulacra, in which the ectoneural cords are internalized into the dermis of the body wall (Figures 2B', 3B, C). The hyponeural cords in all echinoderms always run parallel to the inner surface of the ectoneural system (Figures 2A', B', 3A, A', B).

The primary structural and functional component of the ectoneural and hyponeural systems is a well-developed neuroepithelium. In echinoderms with open ambulacra, the ectoneural neuroepithelium is directly integrated into the epidermis (Figure 2A') being separated from the ambient environment with only a thin apical cuticle (Figure 3A'). In closed ambulacra, the internalized ectoneural neuroepithelium is overlain by a narrow cavity called the *epineural canal*. The outer wall of this canal is formed by a thin *roof epithelium* (Figures 2B', 3B). The internalized ectoneural cords are thus essentially tubular structures. The hyponeural cords are always tubular, with their own cavity, the *hyponeural canal*, between the hyponeural neuroepithelium and the flattened roof epithelium (Figures 2B', 3B).

The ectoneural and hyponeural cords are positioned with the basal surfaces of their respective neuroepithelia facing each other. The two neuroepithelia are connected at intervals by short neural bridges that cross a thin connective tissue partition between the ectoneural and hyponeural systems (Figure 3B).

## 3. Echinoderm radial glia organization and function

For a time, the very existence of glia in echinoderms was questioned (Cobb, 1989). However, a subsequent careful re-examination of the issue with a combination of modern techniques unequivocally demonstrated that glia are indeed present in the echinoderm CNS and play a key role in adult neurogenesis, both under normal conditions and in response to neural injury.

**Figure 4:**
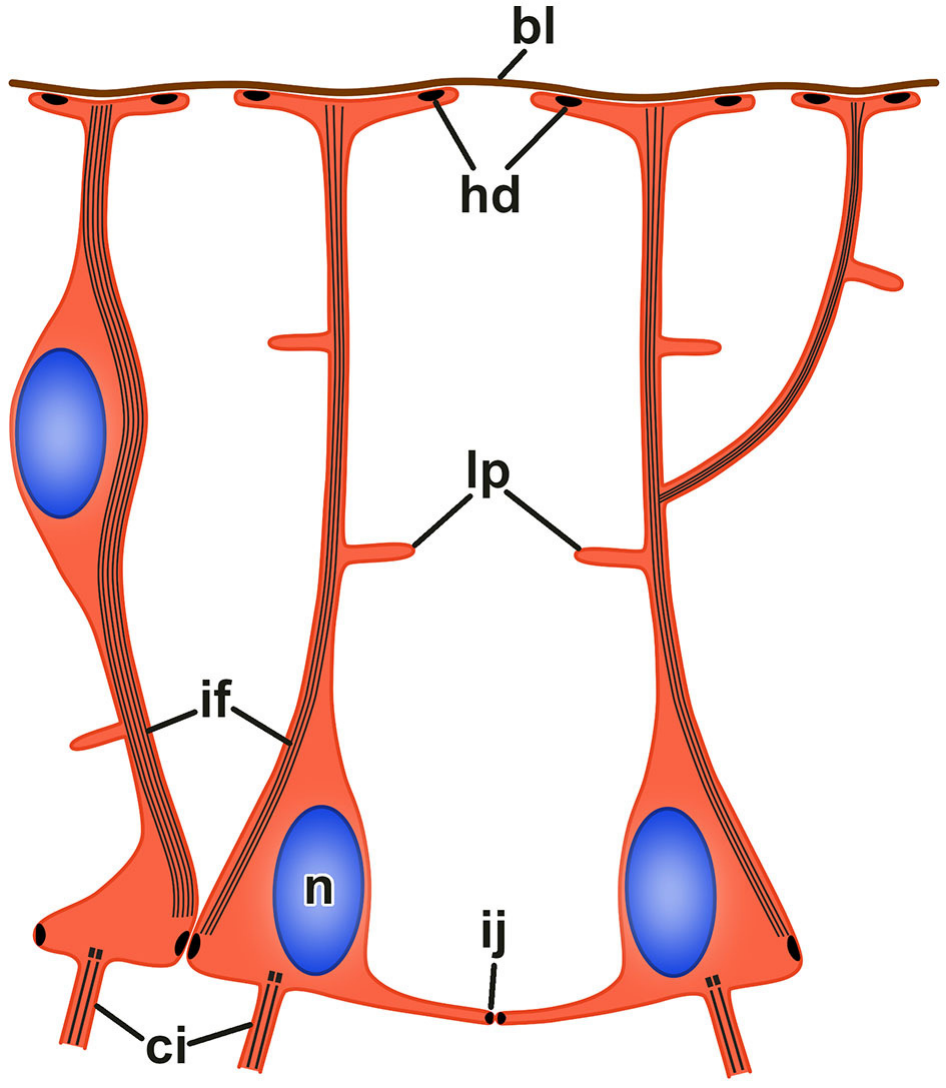
Organization of radial glia in the echinoderm neuroepithelium. *bl*, basal lamina; *ci*, cilium; *hd*, hemidesmosome; *if* , bundle of intermediate filaments; *ij*, intercellular junction; *lp*, lateral protrusion; *n*, nucleus.

The neuroepithelia of the echinoderm CNS contain only one morphologically distinguishable glial cell type (Märkel and Röser, 1991; Viehweg et al., 1998; Mashanov et al., 2006, 2010, 2016; Zueva et al., 2018). These are tall, slender cells that span the height of the neuroepithelium between its apical and basal surfaces (Figures 3A'–C', 4). The cell body containing the nucleus often occupies the apical position. The apical surface bears a cilium that projects into the lumen of the epineural or hyponeural canal. The apicolateral plasma membrane develops intercellular junctions (zonulae adhaerens and septate junctions) that connect adjacent radial glial cells together and, occasionally, to neuronal perikarya. At its basal pole, the cell body of the radial glial cell gives off a long thin process that extends all the way to the basal lamina of the neuroepithleium and attaches to it via hemidesmosomes. These processes contain conspicuous bundles of intermediate filaments (Figure 3C”). They also occasionally bifurcate and bear short side protrusions (Figure 4). Although most of the radial glial cells have their cell bodies at the apical surface of the neuroepithelium, some cells have their nuclei positioned at deeper levels within the neuroepithelium. The cells with the submerged perikarya, however, also span the height of the neuroepithelium and extend two processes, the apical one (directed toward the lumen of the epineural or hyponeural canal) and the basal one (directed toward the basal lamina; Mashanov et al., 2006, 2010).

The epineural and hyponeural roof epithelia are both composed of modified flattened glial cells that also contain short bundles of intermediate filaments in their cytoplasm, but lack basal processes (Märkel and Röser, 1991; Mashanov et al., 2006, 2010, 2016; Zueva et al., 2018).

We determined the ratio of radial glial cells to neurons in the CNS of the sea cucumber *Holothuria glaberrima* (Mashanov et al., 2010). No statistically significant difference in the glia-neuron ratio was found between the ectoneural and hyponeural neuroepithelia of the radial nerve cord (62–69%). However, the relative abundance of glia differed between the radial nerve cords and the nerve ring (~60% vs. 45%). This result suggests the functional differences between the two regions of the CNS. The radial nerve cords are thought to be capable of independently controlling the respective regions of the body (Heinzeller and Welsch, 2001). The role of the nerve ring in the echinoderm CNS is, however, less clear. Some authors think that the nerve ring merely connects the individual radial nerve cords together and relays signals between them (Cobb, 1987). Other workers suggest that the nerve ring contains coordinating centers responsible for coordinating the behavior of the animal as a whole (Smith, 1965).

The body of accumulated data, including ultrastructural observations, immunocytochemistry, cell proliferation, and functional assays, suggest that echinoderm radial glia, the only major glial cell type in the neuroepithelium, is a multifunctional cell type. First, as the only true epithelial cell type in the CNS, radial glia form a supporting scaffold of the neuroepithelium. The features that give radial glia mechanical strength and stability (Bodega et al., 1993; Galou et al., 1997; Zhang and Jiao, 2015) include (a) bundles of intermediate filaments running the length of the cell between their apical and basal surfaces; (b) intercellular junctions that join the adjacent cells together; and (c) the hemidesmosomes that anchor the radial glial cells to the basal lamina. Moreover, the vertical processes of the radial glial cells seem to organize the bundles of nerve processes in the neuropil region of the neuroepithelium (Märkel and Röser, 1991; Viehweg et al., 1998; Mashanov et al., 2006, 2010). Second, the radial glia remove the dying cells and clears the cell debris in the neuroepithelium. This function is particularly evident in the injured CNS, where the phagosomes in radial glial cells are often observed to contain apoptotic bodies (Mashanov et al., 2008). Third, echinoderm radial glial cells exhibit secretory activity. They produce and release a substance that is recognized by antibodies raised against a vertebrate CNS-specific glycoprotein SCO-spondin (Viehweg et al., 1998; Mashanov et al., 2009).

In chordates, SCO-spondin is produced by secretory radial glia in the rostral floor plate and in the subcomissural organ (Guiñazú et al., 2002). This protein performs multiple functions related to the CNS development and maintenance, including: regulation of neurogenesis, neuronal survival and differentiation, axon guidance, prevention of stenosis of the central canal and cerebral aqueduct, sequestering, as well as the transport and regulation of concentration of signaling molecules in the cerebrospinal fluid (Delétage et al., 2021; Sepúlveda et al., 2021). The function of SCO-spondin in echinoderms is currently unknown, but we strive to establish if it has any role in regeneration. Knockdown experiments that become increasingly adapted to a growing cohort of model organisms nowadays should be able to demonstrate that.

### 3.1. Molecular and functional heterogeneity of echinoderm radial glial cells

Based on morphology alone, the radial glial cells in the echinoderm neuroepithelia constitute a single cell type. However, deeper characterization revealed that a range of molecular markers are expressed only in some radial glial cells, but not in others. These results indicate that the echinoderm radial glia is a heterogeneous cell population (Mashanov et al., 2010, 2015b, 2022; Zueva et al., 2018). In some cases, this heterogeneity in molecular signatures has been tied to functional diversity. In other cases, the functional significance behind the expression of the molecular markers remains unknown and still needs to be established with functional genomic techniques.

#### 3.1.1. Calbindin

Some of the radial glial cells of the ectoneural and hyponeural neuroepithelia of the adult sea cucumber CNS were found to display immunoreactivity to antibodies directed against the protein calbindin-d28k (Mashanov et al., 2010). Calbindin-d28k is involved in the regulation of intracellular Ca^2+^ levels. It is abundantly expressed by neurons in most regions of the mammalian brain and helps regulate various calcium-dependent cellular events, including: synaptic plasticity, neurotransmitter release, dendritic spine formation, and neuronal survival (Chard et al., 1995; Meier et al., 1997; Westerink et al., 2012). Although calbindin-d28k is classically known for its role in neurons, it has also been found in some mammalian glial cells, including astrocytes, in which it plays a cytoprotective role after injury (Mattson et al., 1995; Toyoshima et al., 1996; Wernyj et al., 1999). The functional role of calbindin in the echinoderm glia remains to be established.

#### 3.1.2. Brn1/2/4

Brn1/2/4 is an echinoderm ortholog of the vertebrate proteins Brn1, Brn2, and Brn3, which belong to a subgroup (class III) within the POU family of transcription factors (Wolf et al., 2009). In vertebrates, these transcription factors are potent inducers of the neuronal cell fate (Wolf et al., 2009; Vierbuchen et al., 2010). For example, Brn2 was one of the three genes (along with Ascl1 and Myt1l), whose forced expression was used for direct *in vitro* conversion of mouse fibroblasts into functional neurons (Vierbuchen et al., 2010). The important role in the specification of the neuronal fate is evolutionary conserved, as Brn1/2/4 has been shown to be required for differentiation of all post-mitotic neurons in the sea urchin larval nervous system (Garner et al., 2016; Wei et al., 2016). Among adult echinoderms, Brn1/2/4 expression was studied in the brittle star *Ophioderma brevispinum* (Zueva et al., 2018; Mashanov et al., 2022). As expected, this transcription factor was abundantly present in neurons of the radial nerve cord. More surprisingly though, it was also expressed in some, but not all radial glial cells. The role of Brn1/2/4 in the adult echinoderm radial glia still remains largely elusive. What has been established, however, is that this protein prevented the radial glial cells from entering the cell cycle. In the subterminal growth zone near the arm tip (see below), only the Brn1/2/4-negative subpopulation of radial glial cell was capable of undergoing mitotic divisions and producing new cells within the CNS (Mashanov et al., 2022). Similar antimitotic effect was also observed in vertebrates and larval sea urchins, where cells expressing this transcription factor rapidly became post-mitotic before differentiating into neurons (Vierbuchen et al., 2010; Garner et al., 2016).

#### 3.1.3. Myc

Myc proteins are multifunctional transcription factors that control the transcriptional output of thousands of target genes required for a diverse range of biological processes (Patange et al., 2022). One of the prominent roles of Myc genes is their involvement in the regulation of both the embyonic and adult neurogenesis (Cai et al., 2021; Chen and Guan, 2022). Mammals have three Myc paralogs with overlapping functions, c-Myc, n-Myc, and l-Myc, whereas echinoderms have a single Myc gene (Mashanov et al., 2015a).

In the uninjured CNS of the sea cucumber *H. glaberrima*, the transcription factor Myc is abundantly expressed in the apicolateral neurogenic regions (see below) of the ectoneural epithelium of the radial nerve cord. This is the region where most of the radial glial cell bodies are located. At the cellular level, however, not all radial glial cells express Myc. Instead, Myc-positive glial cells are interspersed among Myc-negative cells (Mashanov et al., 2015b). Myc is the only neurogenic gene whose function in adult echinoderm neurogenesis has been directly tested with functional genomic tools (Mashanov et al., 2015c). In the context of the post-traumatic neurogenesis in the holothuroid *H. glaberrima*, we demonstrated that Myc is required for the proper activation of the radial glial cells and for the spike of the programmed cells death during the early post-injury phase of regeneration (Mashanov et al., 2015c). The role of Myc in the activation of neurogenic progenitors and converting them to and maintaining them in the proliferative state appears to be highly conserved in deuterostomes. For example, in the mammalian brain, both c-Myc and n-Myc were shown to be required for the maintenance and function of the neurogenic stem cells (Cai et al., 2021; Chen and Guan, 2022).

## 4. Adult neurogenesis

Two types of adult neurogenesis can be distinguished in echinoderms: (a) neurogenesis in the uninjured adult nervous tissue under “normal” conditions and (b) post-traumatic neurogenesis triggered by an injury or autotomy. Neurogenesis of the first type is a continuous long-term (lifelong) process that results in the replacement of the dead/worn out cells and/or adult growth (e.g., at the tip of the brittle star arm). Neurogenesis of the second type is a relatively short-term event triggered by a CNS injury and aimed at the restoration of the anatomical, histological and functional integrity of the damaged CNS region. Both types of neurogenesis rely on the same source, the radial glial cells, that undergo cell division and give rise to new neuronal and glial cells.

### 4.1. Neurogenesis in the uninjured CNS

Echinoderms are well-known for their ability to rapidly and fully regrow most of their adult tissues and organs, including the central nervous system (Carnevali, 2006). In contrast, generation of new glial and neuronal cells under normal conditions in uninjured animals has received less attention. Nevertheless, the recent recognition of a widespread nature of life-long neurogenesis in many animal taxa (Ganz and Brand, 2016; Kempermann, 2016; Di Cosmo et al., 2018) has prompted a few pilot studies of this phenomenon in echinoderms. As a result, a few key features of the neurogenesis in the intact adult echinoderm CNS have been recently established. First, both neurons and glial cells are continuously eliminated by apoptosis at a slow rate (Mashanov et al., 2010). Second, the lost cells are replenished via continuous proliferation of a subset of radial glial cells (Mashanov et al., 2010, 2013, 2015b). The rate of neurogenesis can outpace the rate of cell loss and lead to a lifelong adult growth (Mashanov et al., 2022). Third, the proliferating radial glial cells are not randomly scattered throughout the CNS, but are located in restricted growth zones.

So far, adult neurogenesis in the uninjured echinoderm CNS has been studied in two species from two different echinoderm subtaxa—the sea cucumber *H. glaberrima* (Mashanov et al., 2015b) and the brittle star *O. brevispinum* (Mashanov et al., 2022). In the sea cucumber, the neurogenic activity is concentrated in the apical zone of the neuroepithelium at the lateral regions of the radial nerve cords (Mashanov et al., 2015b). Some of the newly produced cells leave the place of their origin in the apicolateral neurogenic zones and migrate basally to populate the underlying neural parenchyma (neuropil). The neurogenic zones in the sea cucumber radial nerve cord strongly express many of the same genes that have been previously shown to control various aspects of the adult neurogenesis in vertebrates, including neurogenic stem/progenitor cell maintenance (Hes, Klf1/2/4, Msi1/2, Myc, Oct1/2/11, Piwi, and SoxB1), glial cell fate specification (FoxJ1, Klf1/2/4, and NFI), and neural differentiation (Churchill, ELAV, Lhx1/5, Myc, NeuroD, Prox, and Runt; Mashanov et al., 2015b).

In the brittle star *O. brevispinum*, an active neurogenesis zone was localized in the radial nerve cord at the tip of adult non-regenerating arm (Mashanov et al., 2022). In stellate echinoderms, the arms continuously grow in length throughout the lifespan of the individual (Hotchkiss, 2009, 2012). This adult growth is a continuation of the developmental program. Two structures appear very early in the post-embryonic development: the arm tip and the subterminal growth zone just proximal to it. The subterminal growth zone remains active throughout the subsequent juvenile development and the adulthood and sustains the lifelong extension of the arm via formation of new arm segments. All major anatomical structures of the brittle star arm (e.g., the radial nerve cord, radial water-vascular canal, arm coelom, and epidermis) are represented in the growth zone and each adds new cells from its own pool of progenitor cells (Mashanov et al., 2022). In the radial nerve cord, these dividing progenitors were identified as radial glial cells. As mentioned above, individual radial glial cells differ in their molecular signature. One of the genes that is differentially expressed in subpopulations of the radial glia is the transcription factor Brn1/2/4. It was established that only the Brn1/2/4-negative radial glia in the growth zone acted as the progenitor cell population (Mashanov et al., 2022).

Taken together, the emerging body of data suggests that there are interesting parallels between the neurogenesis in the uninjured echinoderm CNS and adult neurogenesis in vertebrates. One common feature is that the adult neurogenesis is driven by radial glial cells (Alvarez-Buylla and Lim, 2004; Mashanov et al., 2010, 2015b, 2022; Jurisch-Yaksi et al., 2020). The second parallel is that the neurogenesis is confined to certain restricted regions in the CNS. In mammals, these are the subventricular zone and the subgranular zone of the dentate gyrus (Ghosh, 2019). In sea cucumbers, the neurogenic zones are organized as apicolateral longitudinal domains on either side of the radial nerve cord (Mashanov et al., 2015b). In brittle stars, the growth zone is located in the subterminal arm segment (Mashanov et al., 2022). It should be noted here, however, that not all adult vertebrates have clearly restricted neurogenic ares. For example, neurogenesis in teleost fish is more extensive and occurs throughout the spinal cord (S^ırbulescu et al., 2017) and in several dozen brain regions (Zupanc, 2021). Nevertheless, even in the teleostean brain the rate of neurogenesis varies significantly among the neurogenic zones, with the majority (~75%) of new brain cells generated in restricted small areas within cerebellum (Zupanc and Horschke, 1995; Zupanc, 2021). The third common feature between the neurogenesis in vertebrates and echinoderms is the migration of newly generated cells away from the place of their birth (Mashanov et al., 2015b; Kaneko et al., 2017). Finally, the echinoderm neurogenic zones are marked by the expression of the same genetic factors that drive the neurogenesis in vertebrates (Mashanov et al., 2015b,c).

### 4.2. Post-traumatic neurogenesis

The capacity of adult echinoderms to fully regenerate their CNS has long been known (Hyman, 1955). This ability can be easily inferred from simple gross anatomical observations. For example, a fully regenerated arm in stellate echinoderms (i.e., sea stars, brittle stars, and crinoids) contains all its normal tissue components, including the radial nerve cord (Candia Carnevali and Bonasoro, 2001; Biressi et al., 2010; Ben Khadra et al., 2017; Mashanov et al., 2020; Okanishi et al., 2021). Likewise, eviscerating dendrochirotid sea cucumbers discard the entire pharyngeal bulb that contains the nerve ring and anterior ends of all five radial nerve cords. After a few weeks, these anterior CNS regions are fully replaced in the newly regenerated pharyngeal bulb (Kille, 1935; Dolmatov, 1992).

Although anatomically obvious, the post-traumatic neural regeneration had remained relatively unstudied in echinoderms at the cellular and molecular levels until a reproducible and experimentally tractable injury model was established in sea cucumbers (Mashanov et al., 2008, 2013; San Miguel-Ruiz et al., 2009). This injury paradigm involves a complete transection of one of the five radial nerve cords (the mid-ventral radial nerve cord) at the mid-body level and harvesting the tissue samples for cellular and molecular analysis at a series of time points post-injury (Mashanov et al., 2008, 2013; San Miguel-Ruiz et al., 2009). This experimental CNS injury model has yielded most of what we currently know about the cellular and molecular events that are involved in the post-traumatic CNS regeneration in echinoderms.

Immediately after the radial nerve cord transection, the adjacent body wall muscles pull the margins of the wound apart and thus create a wound gap measuring several millimeters wide. The early post-injury phase is marked with a spike of apoptotic cell death in both glial and neuronal populations and an extensive dedifferentiation of radial glial cells in the vicinity of the injury. The dedifferentiating glial cells stop producing and secreting SCO-spondin and lose their long basal processes, which undergo fragmentation and are then phagocytosed by adjacent cells. However, the cell bodies of the dedifferentiating cells maintain their epithelial organization, including the intercellular junctions and apicobasal polarity. Dedifferentiation starts at the site of the injury, then spreads deeper into the regions of the RNC that were not directly impacted by the wound (300–500 μm from the plane of the injury). Both early events, the glial activation and the surge in apoptosis, are controlled by the transcription factor Myc (Mashanov et al., 2015c). Myc expression levels sharply increase immediately after injury and remain upregulated until the regeneration is complete (Mashanov et al., 2015a). Experimental Myc knockdown during the early post-injury period leads to a failure in glial activation and dedifferentiation, as most of the radial glial cells maintain their typical palisade differentiated phenotype. Proper differentiation of the glia is thought to be a pre-requisite for the unfolding of the subsequent glia-driven regeneration events, including generation of new cells and bridging the injury gap (Mashanov et al., 2008, 2013). Another effect of Myc knockdown is a significant reduction in the number of cells undergoing programmed cell death in the vicinity of the injury. The functional significance of the wave of apoptotic cell death at the injury site has not yet been studied. Apoptosis can either be a mere byproduct of the injury, or, as in other animal models (Chera et al., 2009; Vriz et al., 2014), a pre-requisite for regeneration.

Extensive cell death at the injury site during the early phase of regeneration also coincides with a sharp (~50-fold) increase of the expression of the long terminal repeat (LTR) retrotransposon Gypsy1 (Mashanov et al., 2012). The exact role of this mobile genetic element in neural regeneration is unknown, but all glial and neuronal cells expressing the Gypsy1 transcripts avoid apoptotic cell death and contribute to regeneration. In addition, the fact that the expression of specific retroelements correlates with activation of radal glial cells opens avenues for future research into yet unknown mechanisms regulating the plasticity of the radial glial cells. It is still largely unclear how the differential expression of retroelements is regulated in echinoderm regeneration. Nevertheless, some pieces of this puzzle begin to emerge. In particular, in the regenerating arm of the brittle star *O. brevispinum* retrotransposons appear to be activated by the Notch signaling pathway via the double negative regulation mechanism (Mashanov et al., 2020). In this scenario, Notch represses deoxynucleoside triphosphate triphosphohydrolase SAMHD1, a protein known to inhibit endogenous retroelements (Herrmann et al., 2018).

Even though the increase in cell death and glial dedifferentiation both take place simultaneously during the early post-injury phase and both are regulated by Myc, these two cell events are not necessarily coupled. In a recent study (Quesada-Díaz et al., 2021), explants of the radial nerve cord of the sea cucumber *H. glaberrima* were surgically excised and kept *in vitro*. The radial glial cells in the explants started exhibiting signs of dedifferentiation as early as on day three in culture, while the relative abundance of apoptotic cells remained at the basal level. These results indicate that dedifferentiation of surviving glial cells does not necessarily require apoptosis of adjacent cells in the neuroepithelium.

Once dedifferentiated, glial cells become highly proliferative and will give rise to new glial cells and neurons. After radial nerve cord transection in sea cucumbers (Mashanov et al., 2008, 2013), the activated glial cells of the ectoneural and hyponeural neuroepithelia on either side of the wound form separate tubular rudiments. These epithelial tubes seal the cut distal surface and thus end blindly at their leading tips. Their central lumen is continuous with the epineural or hyponeural canals of the radial nerve cord stump, respectively. The epithelial walls of the tubular outgrowth are composed of irregularly shaped radial glial cells that often develop filopodium-like extensions protruding into the surrounding extracellular matrix. Neuronal cell bodies and processes are mostly absent in these early rudiment. The glial tubes grow toward each other through the connective tissue that fills the wound gap. Dividing glial cells are observed along the entire length of the rudiment, indicating that the growth is accomplished via intercalation of new cells, rather than addition of new cells at the leading tip. Eventually, the anterior and posterior glial tubes fuse to restore the anatomical continuity of the radial nerve cord. The ectoneural rudiments grow faster and fuse earlier than the hyponeural cords. The basal region of the neuroepithelia becomes populated by neuronal cell bodies and their processes making the neuroepithelium to progressively thicken. The newly born neurons were found to persist long-term (i.e., at least for 133 days in the longest tracking experiment, until it was stopped; Mashanov et al., 2013). These neuronal cells express molecular markers typical of mature echinoderm neurons, and establish synaptic connections. These observations indicate the functional integration of new neuronal cells into the CNS neural circuits. The newly regenerated segment of the radial nerve cord gradually differentiates and fully restores its normal tissue organization so that it eventually becomes indistinguishable from the non-injured regions of the radial nerve cord.

It is interesting to note the similarities between the neurogenesis in the uninjured CNS and post-traumatic neural regeneration in echinoderms. The most interesting parallel is that both phenomena rely on radial glial cells as the progenitor population. This observation is in line with the growing body of evidence that suggest that the capacity for post-traumatic adult regeneration strongly correlates with indeterminate growth and that the mechanisms underlying the normal growth can be co-opted in regeneration (Vogt, 2012; Hariharan et al., 2016). Better understanding of the relationships between these two phenomena in basic studies can be harnessed in the clinic to repair spinal cord and brain injury in human patients. Humans undergo limited adult neurogenesis. It has been shown that traumatic brain injuries increase the rate of the new cell generation in the two continuously active mammalian neurogenic zones, the dentate gyrus and the subventricular zone, and activate quiescent neural progenitors in other CNS regions. However, this intrinsic regenerative response falls short of fully restoring the damaged tissue structure and function. Further comparative studies of animal models that are capable of both normal physiological neurogenesis and efficient post-traumatic CNS repair will provide insights into how the limited neurogenic potential in humans can be improved to fully address the therapeutic goals via novel approaches to neural regeneration therapies.

## 5. Conclusions

- The echinoderm radial glia shares a number of key characteristics with the radial glia of chordates, including:

- the orthogonal orientation relative to the surface of the neuroepithelium;
- the slender elongated shape spanning the height of the neuroepithelium;
- the epithelial nature with clear apicobasal cell polarity and apical cilia;
- the well-developed cytoskeleton composed of bundles of intermediate filaments in the cytoplasm;
- the ability to produce and secrete SCO-spondin;
- the requirement of Myc expression for the activation of the neural progenitor function.

- The neurogenic progenitor population in echinoderm neurogenesis is a subset of radial glial cells. The neurogenic activity in these cells is known to be defined by the differential activity of two transcription factors: the expression of Myc and the absence of Brn1/2/4. Myc is required for the glia activation in response to injury. Brn1/2/4 needs to be absent in order for the glia to be able to function as the progenitor cell population.
- The morphological, molecular, and functional similarities between the echinoderm radial glia and their counterparts in chordates reveals that radial glia is a phylogenetically ancient cell type that would have emerged at least in the last common ancestor of ambulacrarians and chordates. The stereotypical role of the radial glia in neurogenesis raises the possibility that the fundamental mechanisms of neurogenesis are conserved in deuterosomes. Further research into the neurogenesis of highly regenerative echinoderms will help us understand how the latent phylogenetically conserved cellular and molecular mechanisms can be harnessed to develop new efficient treatment options for human CNS injuries.

## Author contributions

VM: Conceptualization, Writing—original draft. SA: Writing—review and editing. DJM: Writing—review and editing. RR: Writing—review and editing. DJ: Writing—review and editing.
